# Electroacupuncture Involved in Motor Cortex and Hypoglossal Neural Control to Improve Voluntary Swallowing of Poststroke Dysphagia Mice

**DOI:** 10.1155/2020/8857543

**Published:** 2020-09-27

**Authors:** Shuai Cui, Shuqi Yao, Chunxiao Wu, Lulu Yao, Peidong Huang, Yongjun Chen, Chunzhi Tang, Nenggui Xu

**Affiliations:** ^1^South China Research Center for Acupuncture and Moxibustion, Medical College of Acupuncture Moxibustion and Rehabilitation, Guangzhou University of Chinese Medicine, 510006 Guangzhou, China; ^2^Research Institute of Acupuncture and Meridian, Anhui University of Chinese Medicine, 230038 Hefei, China; ^3^Acupuncture Massage and Rehabilitation Institute, Yunnan University of Chinese Medicine, 650500 Kunming, China

## Abstract

The descending motor nerve conduction of voluntary swallowing is mainly launched by primary motor cortex (M1). M1 can activate and regulate peripheral nerves (hypoglossal) to control the swallowing. Acupuncture at “Lianquan” acupoint (CV23) has a positive effect against poststroke dysphagia (PSD). In previous work, we have demonstrated that electroacupuncture (EA) could regulate swallowing-related motor neurons and promote swallowing activity in the essential part of central pattern generator (CPG), containing nucleus ambiguus (NA), nucleus of the solitary tract (NTS), and ventrolateral medulla (VLM) under the physiological condition. In the present work, we have investigated the effects of EA on the PSD mice *in vivo* and sought evidence for PSD improvement by electrophysiology recording and laser speckle contrast imaging (LSCI). Four main conclusions can be drawn from our study: (i) EA may enhance the local field potential in noninfarction area of M1, activate the swallowing-related neurons (pyramidal cells), and increase the motor conduction of noninfarction area in voluntary swallowing; (ii) EA may improve the blood flow in both M1 on the healthy side and deglutition muscles and relieve PSD symptoms; (iii) EA could increase the motor conduction velocity (MCV) in hypoglossal nerve, enhance the EMG of mylohyoid muscle, alleviate the paralysis of swallowing muscles, release the substance P, and restore the ability to drink water; and (iv) EA can boost the functional compensation of M1 in the noninfarction side, strengthen the excitatory of hypoglossal nerve, and be involved in the voluntary swallowing neural control to improve PSD. This research provides a timely and necessary experimental evidence of the motor neural regulation in dysphagia after stroke by acupuncture in clinic.

## 1. Introduction

“Dysphagia” is defined as an obstacle to the flow of liquids/pills from the mouth to the esophagus. Dysphagia is a serious problem in various neurologic diseases, and it is associated with an increase in morbidity and mortality [1–6]. Stroke is the most common neurologic cause of dysphagia. Severe dysphagia is usually observed during the first 2–4 weeks after stroke, and a prevalence of 29%–81% has been documented. However, minor disorders of swallowing have been reported at a prevalence of 91% in the stroke patients [5, 7–9]. Dysphagia can result in significant complications, such as malnutrition, aspiration pneumonia, and poor quality of life [10–12]. A large number of functional and structural abnormalities are present in dysphagia patients in each of its phases, such as the oral cavity, pharynx, larynx, or esophagus [13–15].

To initiate and regulate swallowing, a combination of feedback and motor planning is required [16]. Most scholars believe that the cortical swallowing center is concentrated mainly in the primary sensorimotor cortex, anterior cingulate gyrus, and insula [17]. The characteristics of injury to the cortical swallowing center are multifocal and bilateral, damage to which can affect the subthreshold excitability of nucleus tractus solitarii and nucleus ambiguous in the central pattern generator (CPG) for swallowing and reduce its regulation of swallowing function [18]. M1 can regulate the processing and transmission of neural information in the brain. Yuan and his colleagues found that the excitability and area of the motor cortex associated with swallowing were increased in the left hemisphere and alleviated the swallowing dysfunction of patients if the right motor cortex was injured [19]. That result showed that dysphagia improvement required maintenance of bilateral pathways. However, most studies mainly involved in healthy people or a small sample of patients with dysphagia, while experimental studies exploring the underlying mechanisms are so limited. Therefore, further research is required to understand how the motor cortex conducts through the descending motor nerves, affects swallowing function, and how blood flow changes in the brain and deglutition-muscle groups after dysphagia occurring.

The life quality of the patients suffering from poststroke dysphagia (PSD) can be affected severely if the PSD is caused by cortical ischemic injury, but various early rehabilitation programs can improve this situation [20]. Interventions for patients with dysphagia include electromyographic biofeedback, Mendelsohn maneuver, repetitive transcranial magnetic stimulation, and surface neuromuscular electrical stimulation. The mechanism underpinning neural regulation of swallowing is poorly understood. Hence, the relevant treatment and research methods are limited to only local changes in neural regulation of swallowing function, and the efficacy of some treatment methods has not been demonstrated. However, as a conventional therapy for stroke rehabilitation in China, acupuncture has been used extensively as a complementary or alternative therapy worldwide [21]. In PSD patients with cortical hemisphere injury, several studies have suggested that acupuncture may be helpful for patients' recovery [22, 23]. Animal experiments have shown that electrical stimulation can induce continuous swallowing [24]. In previous work, we have demonstrated that electroacupuncture (EA) could regulate swallowing-related motor neurons and promote swallowing activity in the essential part of central pattern generator (CPG), containing nucleus ambiguus (NA), nucleus of the solitary tract (NTS), and ventrolateral medulla (VLM) under the physiological condition ([25]; You H et al., 2018; [26]). Some researchers have also found that electrical stimulation can reorganize the motor-cortex neurons involved in swallowing. Only M1, thalamus, and insula are activated after transcutaneous electrical stimulation in pharyngeal muscle; in addition, cortical recombination mediated by electrical stimulation improves swallowing function, which is closely correlated to the occurrence and parameters of electrical stimulation [27–29]. M1 has a crucial role in the neural pathway that controls voluntary swallowing. Although its role in the voluntary oral phase of swallowing is undisputed [30], its precise role in motor control of the pharyngeal phase is not clearly defined, and the descending neural regulation of swallowing is also not clear.

In the present study, the mice suffering swallowing dysfunction after stroke were treated by EA. We aimed to observe the descending motor nerve regulation mechanism and blood flow changes involved in voluntary swallowing, so we could further illuminate how EA intervene the motor neural control of voluntary swallowing to improve PSD in mice.

## 2. Materials and Methods

### 2.1. Ethical Approval of the Study Protocol

This study was carried out in accordance with the principles of the Basel Declaration and recommendations of the guidelines of the Guangzhou University of Chinese Medicine Committee for Care and Use of Research Animals. The protocol was approved by the Guangzhou University of Chinese Medicine Committee for Care and Use of Research Animals.

### 2.2. Animals

Animals were provided by the Animal Laboratory Center of Guangzhou University of Chinese Medicine (Guangzhou, China; experimental animal certificate number: 44005800008103; animal license number: SCXK (Yue) 2013-0034).

Male C57BL/6J mice (8 months, specific pathogen-free, 25–33 g) were housed in individual cages under standard laboratory conditions. Food and water were supplied ad libitum.

A total of 12 mice from 67 mice were randomly selected as a normal group to test laser speckle contrast imaging (LSCI), electroencephalogram (EEG), electromyography (EMG), water intake, and substance P (SP) concentration.

The rest 55 mice were used to build a PSD model by photochemical method. EA and sham EA were applied on the PSD model. For EA, the needle was retaining, and electric stimulation lasted 15 min, but for sham EA, no electric stimulation was given. EA (acute) was only stimulated for one day; EA (chronic) was stimulated continuously for three days. 18 mice were randomly selected into three groups: sham EA, EA (acute), and EA (chronic) to test LSCI and electrophysiology recording, which were compared before and after stimulation. 24 mice randomly selected four groups: model, sham EA, EA (acute), and EA (chronic) to test electromyography (EMG), water intake, and SP concentration. 7 mice from 67 mice were excluded because the model was not successful.

### 2.3. PSD Model

We referred to the modeling method of Michael Schroeter [31]. Mice were fixed with a holder. Tail veins were injected with Rose Bengal solution (1.5%; Sigma–Aldrich, Saint Louis, MO, USA) at 10 *μ*L/g body weight. After injection, anesthesia was induced with 4% isoflurane. Then, anesthesia was maintained with 2% isoflurane using a mask.

**Figure 1:**
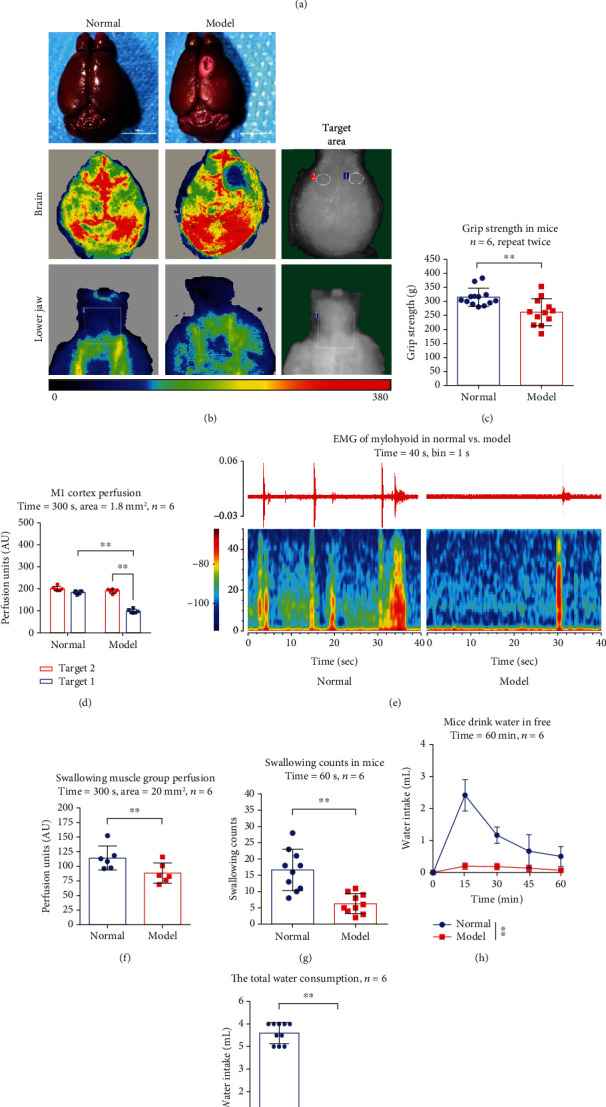
Evaluation of some indexes between the poststroke dysphagia (PSD) model and normal mice. (a) The PSD model process and cerebral infarcts position. (b) Laser speckle contrast imaging (LSCI): the brain and lower jaw in normal and the model group. (c) Grip strength test: normal vs. model: ∗∗*p* < 0.01. (d) Motor cortex perfusion: time = 300 s, target 1 = noninfarction, target 2 = infarction, target area = 1.8 mm^2^, normal vs. model (target 1): ∗∗*p* < 0.01, target 2 vs. target 1 (model): ∗∗*p* < 0.01. (e) Electromyography (EMG) of mylohyoid and real-time spectrum in normal vs. model group, time = 40 s, bin = 1 s. (f) Swallowing muscle perfusion: time = 300 s, target area = 20 mm^2^, normal vs. model: ∗∗*p* < 0.01. (g) Swallowing counts: time = 60 s, normal vs. model: ∗∗*p* < 0.01. (h) Drink water at different times: time = 0 min, 15 min, 30 min, 45 min, and 60 min. Normal vs. model: ∗∗*p* < 0.01. (i) Total water consumption: normal vs. model: ∗∗*p* < 0.01.

Mice were fixed on stereotaxic apparatus (RWD Biotechnology, Shenzhen, China). The skull was exposed, and the correct M1 coordinates were located (1 mm lateral and 0.16 mm posterior to the bregma; depth from the brain surface, 1 mm). A laser (wave length: 530 nm; power: 15 mW) was used to irradiate an area of ~2 mm^2^. After 8 min of irradiation, the scalp was sutured, and mice were placed back in their cages to recover from anesthesia. Swallowing-related muscle function and water intake were evaluated in conscious mice (Figures 1(a) and 1(g)–1(i)).

### 2.4. EA Parameters

**Figure 2:**
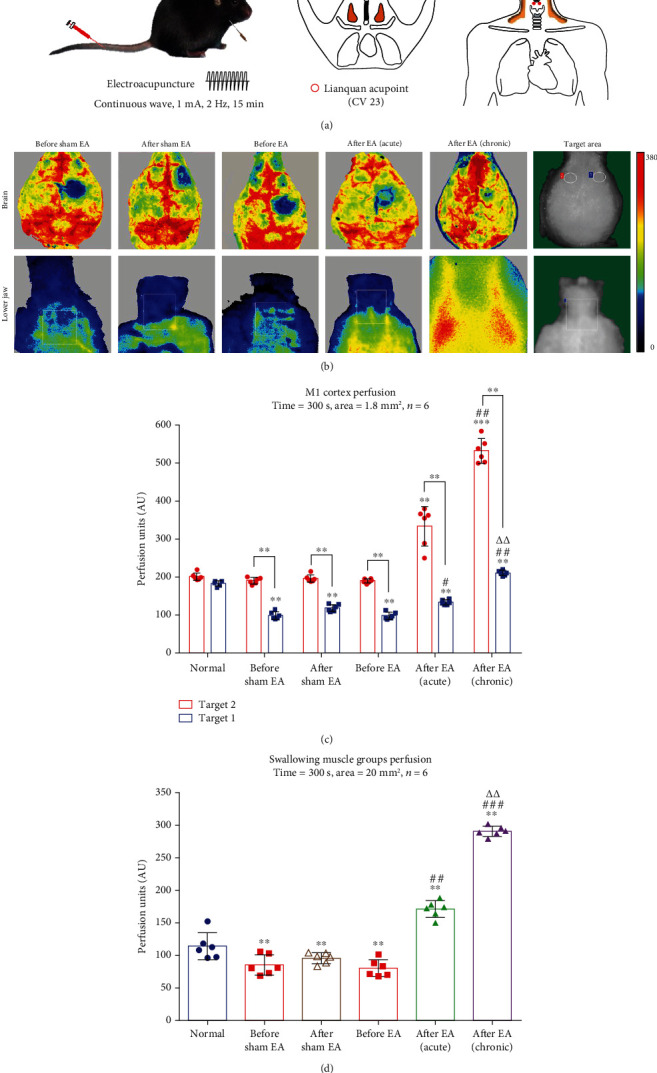
The brain and lower jaw blood perfusion changes at before and after electroacupuncture in the PSD model.(a) Electroacupuncture parameters: continuous wave, 1 mA, 2 Hz, and 15 min. Acupoint: Lianquan (CV 23). (b) The brain and lower jaw of LSCI in each group: sham EA = lack of electric stimulation, after EA (acute) = EA stimulation once, and after EA (chronic) = EA stimulation for 3 days. (c) Motor cortex perfusion: time = 300 s, target 1 = noninfarction, target 2 = infarction, target area = 1.8 mm^2^, normal vs. others group (target 1): ∗∗*p* < 0.01. Target 2 vs. target 1: ∗∗*p* < 0.01 in others group except normal. Before EA vs. after EA (acute) (target 1): ^#^*p* < 0.05; before EA vs. after EA (chronic) (target 1): ^##^*p* < 0.01. Before EA vs. after EA (acute) (target 2): ∗∗*p* < 0.01, before EA vs. after EA (chronic) (target 2): ∗∗∗*p* < 0.001. After EA (acute) vs. after EA (chronic) (target 2): ^##^*p* < 0.01. After EA (acute) vs. after EA (chronic) (target 2): ^△△^*p* < 0.01. (d) Swallowing muscle perfusion: time = 300 s, target area = 20 mm^2^, compared with normal, ∗∗*p* < 0.01 in others group. Before EA vs. after EA (acute): ^##^*p* < 0.01. Before EA vs. after EA (chronic): ^###^*p* < 0.001. After EA (acute) vs. after EA (chronic): ^△△^*p* < 0.01.

Anesthesia was induced using 4% isoflurane. Mice were laid supine and fixed. Anesthesia was maintained with 2% isoflurane using a mask. First routinely sterilized neck skin, then we located CV23. We inserted an acupuncture needle to the upper margin of the midline of the mandible. Another needle was inserted 2 mm adjacent to CV23. The needling depth of CV23 is 5 mm. An EA apparatus (continuous wave; current, 1 mA; frequency, 2 Hz; time, 15 min per day; HANS-200A/100B; HANS, Beijing, China) was attached to the acupuncture needle: acute EA treatment for 1 day and chronic EA treatment for continuous 3 days (Figure 2(a)).

### 2.5. Grip Strength Test

The grasping power of mice limbs was measured by a grip tester (YLS-13Al; YiyanTechnology & Development, Shandong, China). Mice of identical age were employed in this experiment only if their toes were not damaged.

Mice were placed carefully on a grip power board. Their tails were held, and mice were pulled back gently in the horizontal direction. The mice were pulled back with even greater force after they grasped the plate, causing them to loosen their claws. The instrument recorded the maximum grip strength of mice limbs automatically. This experiment was repeated twice for each mouse in each group, and the average value was taken.

### 2.6. LSCI

Mice were anesthetized with 2% isoflurane and then fixed on stereotaxic apparatus (RWD Biotechnology). The skull was exposed. A laser was focused on the target area. Recording was lasted for 5 min. We observed changes in total cerebral blood flow to selected infarction area and noninfarction area (area: 1.8 mm^2^) of the motor cortex and compared blood perfusion changes in each group.

Mice were laid supine and fixed. Fur near the mylohyoid muscle was removed by using depilatory cream. The laser was focused on the swallowing muscles (area: 20 mm^2^). Recording was lasted for 5 min. We observed the blood perfusion changes around the swallowing muscles.

### 2.7. Recording of the Discharge of M1 Neurons *In Vivo*

Anesthesia was maintained with 2% isoflurane, and mice were fixed on stereotaxic apparatus. The skull was exposed after routine sterilization. The left M1 coordinates were located (bregma: −0.16 mm; LR: 1 mm; H: 1 mm). Then, 2 × 4 + 1 matrix electrodes were implanted in the target brain region to observe spontaneous discharges through a multichannel recording system (Plexon, Dallas, TX, USA). Spikes and LFP were recorded for 5 min in each group. An offline sorter (Plexon) was used to filter signals. Processed signals were analyzed statistically using NeuroExplorer™ (Nex Technologies, Lexington, MA, USA).

### 2.8. Recording of Hypoglossal Nerves *In Vivo*

**Figure 3:**
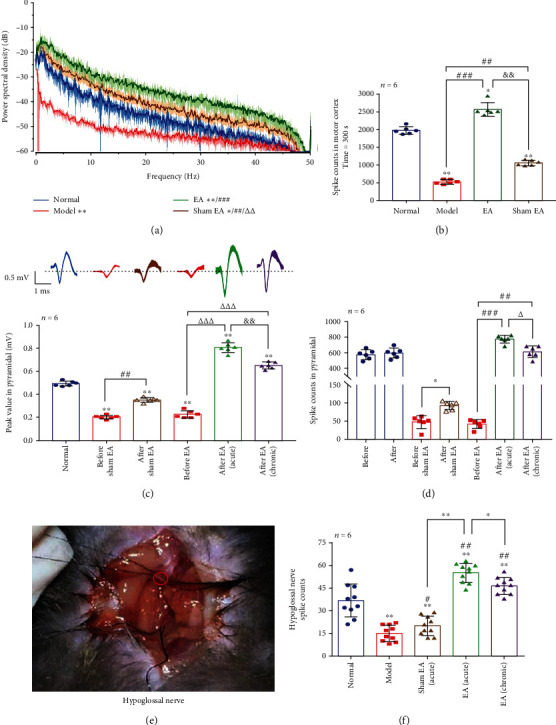
Electroacupuncture stimulation affects central and peripheral neurons activities. (a) Power spectral density in each group. Normal vs. model, ∗∗*p* < 0.01; normal vs. EA, ∗∗*p* < 0.01; normal vs. sham EA, ∗*p* < 0.05. Model vs. EA, ^###^*p* < 0.001; model vs. sham EA, ^##^*p* < 0.01. EA vs. sham EA, ^△△^*p* < 0.01. (b) Spike counts in motor cortex: time = 300 s, compared with normal, ∗*p* < 0.05 in EA and ∗∗*p* < 0.01 in the model and sham EA. Compared with the model, ^###^*p* < 0.001 in EA and ^##^*p* < 0.01 in sham EA. Compared with EA, ^&&^*p* < 0.01 in sham EA. (c) Pyramidal peak value in each group: compared with normal, ∗∗*p* < 0.01 in others group. Before sham EA vs. after sham EA: ^##^*p* < 0.01. Before EA vs. after EA (acute): ^△△△^*p* < 0.001, before EA vs. after EA (chronic): ^△△△^*p* < 0.001. After EA (acute) vs. after EA (chronic): ^&&^*p* < 0.01. (d) Pyramidal spike counts in each group: before sham EA vs. after sham EA: ∗*p* < 0.05, before EA vs. after EA (acute): ^###^*p* < 0.001, before EA vs. after EA (chronic): ^##^*p* < 0.01, and after EA (acute) vs. after EA (chronic): ^△^*p* < 0.05. (e) Photograph was shown that hypoglossal nerve has been separated. (f) Hypoglossal nerve spike counts: compared with normal, ∗∗*p* < 0.01 in others group. Compared with model, ∗*p* < 0.05 in sham EA (acute) and ∗∗*p* < 0.01 in EA (acute) and EA (chronic). Compared with sham EA (acute), ∗∗*p* < 0.01 in EA (acute). Compared with EA (acute), ∗*p* < 0.05 in EA (chronic).

Mice were fixed supine. Anesthesia was maintained with 2% isoflurane. The cervical hypoglossal nerve was isolated carefully and then drawn out with a thin wire (Figure 3(e)). Bipolar platinum recording electrodes were hooked up to the hypoglossal nerve. A drop of paraffin oil was placed over the surface of the nerve. The reference electrode was inserted into nearby subcutaneous tissue. After waking from anesthesia, water feeding was initiated with a 5 mL microsyringe. Changes in activity of hypoglossal nerve was evoked and recorded by Spike2 in mice swallowing (Fig. S4).

### 2.9. EMG Recording in the Mylohyoid Muscle *In Vivo*

Anesthesia was induced with 4% isoflurane. Mice were fixed supine on stereotaxic apparatus at 45°. Pure water (4 mL) was extracted with a 5 mL intragastric syringe and placed in a microinjection pump (Stoelting, Wood Dale, IL, USA). The angle of the lavage needle was adjusted for placement under the tongue. A recording electrode was inserted into the mylohyoid muscle. The reference electrode was inserted into the masseter muscle. After waking up from anesthesia, mice were given water (2 *μ*L/s, 5 s). The EMG activity of the mylohyoid muscle was evoked and recorded by the Spike2 software (CED, Cambridge, UK) when mice were swallowing water.

### 2.10. MCV

**Figure 4:**
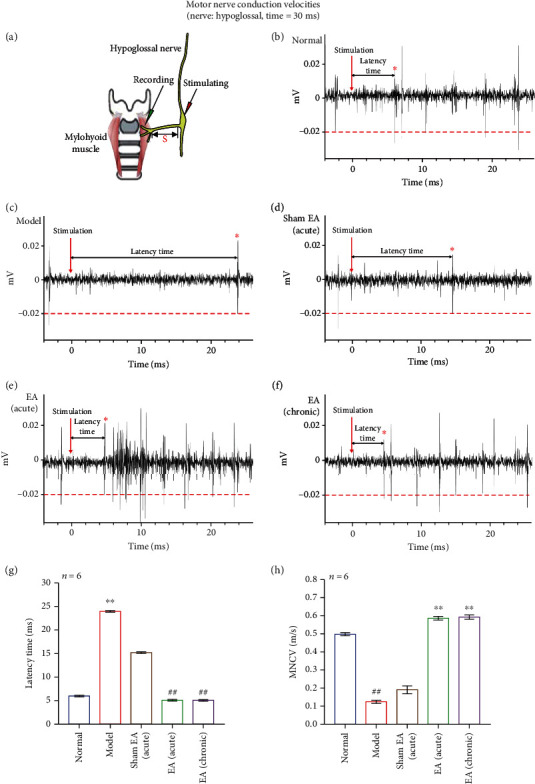
Motor conduction velocity of hypoglossal nerve. (a) Stimulation electrode: hypoglossal nerve trunk; recording electrode: mylohyoid muscle; the distance between nerve trunk and muscle: *s*; the latency between stimulation point and the first EMG evoked by mylohyoid muscle: *t*; MCV: *v* = *s*/*t*. (b–f) The latency between the stimulation point and the first induced EMG (marked with an asterisk) in each group. Model: PSD; sham EA: acupuncture without electric; EA acute: EA for 1 day; EA chronic: EA for 3 days. MCV was detected after treatment in each group. (g) Difference of latency in each group. Compared with the normal, the model increased significantly (∗∗*p* < 0.01); compared with the model, EA (acute) and EA (chronic) have obviously decreased (^##^*p* < 0.01). (h) Difference of MNCV in each group. Compared with the normal, the model increased significantly (^##^*p* < 0.01); compared with the model, EA (acute) and EA (chronic) have obviously decreased (∗∗*p* < 0.01).

Mice were placed under 2% isoflurane anesthesia in the supine position. One side of the hypoglossal nerve was separated, and the mylohyoid muscle was exposed. The hook-shaped stimulation electrode was placed in the hypoglossal nerve trunk, the recording electrode was placed in the mylohyoid muscle, and the reference electrode was placed in the nearby tissue. Pulling the hook electrode of hypoglossal nerve trunk for 1 second, the evoked EMG was observed (threshold: 0.02 mV, duration: 30 ms). The time from the stimulation point to the first evoked action potential was the latency: *t*, the distance from the nerve trunk to the mylohyoid muscle: *s*, and conduction velocity: *v* = *s*/*t* (Figure 4(a)).

### 2.11. Water Intake

Five groups of mice (*n* = 6) were fed separately and were deprived of water for 1 day. On day 2, each group was given drinking water *ad libitum*, and we recorded the changes in water intake at 15, 30, 45, and 60 min, as well as the total water intake in 1 day.

### 2.12. SP Concentration in Serum

Blood was obtained from the eyeballs of mice in each group after relevant experimental recording was completed. The extracted blood was placed at room temperature for 2 h and centrifuged at approximately 3500 × g for 15 min (at 4°C or room temperature for prechilled samples). After centrifugation, the supernatant was collected. The SP concentration was measured using an ELISA kit (ENZO Life Sciences, Farmingdale, NY, USA). Changes in the serum SP concentration in each group were compared.

### 2.13. Linear Correlation Analysis

Linear regression analysis was carried out on swallowing counts, water intake, SP concentration, swallowing-related pyramidal cell spike counts, and hypoglossal nerve spike counts.

### 2.14. Statistical Analyses

Statistical analyses were undertaken using SPSS 23.0 (IBM, Armonk, NY, USA). The difference between groups was analyzed by one-way ANOVA. The homogeneity of the variance was tested before comparisons between groups were made. The least-square difference test was used for homogeneity of variance, and the Tamhane's T2 test for heterogeneity of variance. *p* < 0.05 was considered as statistically significant.

M1 neurons were analyzed by Offline Sorter™ and NeuroExplorer. Analyze methods including Raster, PCA, autocorrelation, and power spectral density were used.

The method of identifying neurons in M1 through autocorrelation analysis [32] and the characteristics of pyramidal cells were as follows: (1) low mean discharge frequency (0.5–10 Hz) and irregular discharge pattern; (2) the ISI histogram showed that the short ISI (3–10 ms) was dominant, and exponential attenuation was present after 3–5 ms ISI; and (3) wide waveform (>300 *μ*s). The characteristics of interneurons were as follows: (1) high mean discharge frequency (>5 Hz); (2) the ISI histogram presented delayed spikes and slower attenuation; and (3) narrow waveform (<250 *μ*s).

## 3. Results

### 3.1. Impaired Blood Flow and Swallowing Function in PSD Mice Model

To induce the PSD model, we used the photochemical method to cause infarction area in right M1, then to evaluate the model (Figure 1(a)). Compared with the control group, the grip power in the model decreased significantly in the grip strength test (*n* = 6 mice/group, *p* < 0.01), which suggested that symptoms of stroke caused by ischemia in the motor cortex (Figure 1(c)). By observing the brain and the lower jaw with laser speckle contrast imaging (LSCI), we found the right of M1 showed an obvious focal ischemia, and the blood perfusion of lower jaw was lower in the model (Figure 1(b)). Compared the target 1 (infarction area) with the target 2 (noninfarction area), the M1 perfusion was significantly different (*n* = 6 mice/group, *p* < 0.01). Compared with normal, the target 1 of the model was obviously decreased (*n* = 6 mice/group, *p* < 0.01), and swallowing muscle perfusion was significantly decreased (*n* = 6 mice/group, *p* < 0.01) (Figures 1(d) and 1(f)), which suggested that the blood flow in the brain was changed in the PSD model. Observing the EMG of mylohyoid in the model, we found that the real-time power spectrum and swallowing counts were reduced (*n* = 6 mice/group, *p* < 0.01) (Figures 1(e) and 1(g)), and water intake and total water consumption were also decreased, compared with the control group (*n* = 6 mice/group, *p* < 0.01) (Figures 1(h) and 1(i)). These results indicate swallowing and water intake dysfunction were caused by the PSD model.

### 3.2. Effect of EA at CV23 on Cerebral Blood Flow and Lower Jaw in PSD Mice

LSCI showed different cerebral blood flow and lower jaw at before and after EA or sham EA change (Figure 2(b)). The blood flow showed significant difference between infarction (target 1) and noninfarction (target 2) group (*n* = 6 mice/group, *p* < 0.01). After EA (acute), blood perfusion was increased in the noninfarction area (*n* = 6 mice/group, *p* < 0.01), and after EA (chronic), it was significantly increased, compared to before EA (*p* < 0.001) and after EA (acute) group (*p* < 0.01). These results illustrate EA could promote the blood perfusion of M1 in the noninfarction area. The infarction area of M1 perfusion showed significant difference between each group (*n* = 6 mice/group, *p* < 0.01). After EA (acute) was increased than before EA in the infarction area (*n* = 6 mice/group, *p* < 0.05); after EA (chronic) was significantly increased than before EA (*p* < 0.01) and after EA (acute) (*p* < 0.01) (Figure 2(c)). These results show EA could supply blood perfusion in the infarction area and alleviate the infarction area.

In the lower jaw, swallowing muscle perfusion showed significant difference between each group (*n* = 6 mice/group, *p* < 0.01). Compared with before EA, the blood perfusion of after EA (acute) was increased (*n* = 6 mice/group, *p* < 0.01), after EA (chronic) was dramatically increased (*p* < 0.001); compared with after EA (acute), after EA (chronic) was significantly increased (*p* < 0.01) (Figure 2(d)). These results demonstrate EA could improve the blood flow of the lower jaw.

### 3.3. The Neurons in Noninfarction Area of M1 Were Activated by EA in PSD Mice

We implanted the multichannel electrodes into the noninfarction area of M1, compared spike counts in each group at before and after condition (Fig. S3A, S3B). First, we observed the normal group (*n* = 6) at before and after recording: in total 5 units, 2 units were defined as interneurons, and 3 units were defined as pyramidal cells (Fig. S1A, S2A). Before vs. after, spike continuous (SPKC) and local filed potential (LPF) showed no difference (before: interneurons account for 70.35%, and pyramidal cells account for 29.65%; after: interneurons account for 64.90%, and pyramidal cells account for 35.10% (Fig S3D)). In addition, we stimulated the model group (*n* = 6) with sham EA (lack of electric). In total recording 3 units, 2 units were interneurons, and 1 unit was pyramidal cell (Fig. S1B , S1C; Fig. S2B, S2C). Before sham EA vs. after sham EA, SPKC and LFP were obviously decreased (before sham EA: interneurons account for 82.99%, and pyramidal cells account for 17.01%; after sham EA: interneurons account for 81.75%, and pyramidal cells account for 18.25% (Fig. S3E)). Third, we stimulated the model group (*n* = 6) with EA (acute: 1 day): recording 3 units, 2 units were interneurons, and 1 unit was pyramidal cell (Fig. S1D, S2D). Before EA vs. after EA, SPKC and LFP were obviously increased. Before EA, interneurons account for 84.27%, and pyramidal cells account for 15.73%; after EA (acute), interneurons account for 66.38%, and pyramidal cells account for 33.62% (Fig. S3F). Finally, we sustained EA (chronic: 3 days) to stimulate the model group: recording 3 units, 2 units were interneurons, and 1 unit was pyramidal cell (Fig. S1E, S2E). After EA (acute) vs. after EA (chronic), SPKC and LFP were relatively stable. After (chronic), interneurons account for 65.40%, and pyramidal cells account for 34.60% (Figure S3F). These results indicate EA could evoke noninfarction neurons activities; the effect of chronic EA was lasting and stable than acute EA.

In the power spectral density, compared with the model, sham EA was different (*p* < 0.01); EA was significantly different (*p* < 0.001); compared with sham EA, EA was different (*p* < 0.01). The results show that EA increased the energy of LFP (Figure 3(a)). At the same time, we found that the total spike counts of each group in M1 (*n* = 6, time = 300 s) were also changed; model was obviously decreased than normal (*p* < 0.01), but after EA, the spike counts were significantly increased (*p* < 0.001); it shows that EA can promote population neuronal activity of M1 in PSD mice (Figure 3(b)).

We identified the number of pyramidal cells in the motor cortex and observed their peak value and spike counts. The number of pyramidal cells in each group (*n* = 6) was normal = 15, model = 2, sham EA = 4, EA (acute) = 8, and EA (chronic) = 10 (Table 1). Compared with peak value at before and after in each group (0.5 mV, 1 ms, *n* = 6), each group was significantly different, compared with normal (*p* < 0.01); after sham EA of peak value and spike counts were increased than before sham EA (*p* < 0.01 and *p* < 0.05, separately); after EA (acute) and after EA (chronic) of peak value and spike counts were obviously increased than before EA (*p* < 0.001 and *p* < 0.01, separately); after EA (chronic) of peak value and spike counts were decreased than after EA (acute) (*p* < 0.01 and *p* < 0.05, separately). These demonstrated that EA could promote the potential intensity of pyramidal cells, chronic EA flattens the potential intensity of them (Figures 3(c) and 3(d)).

### 3.4. The Effects of EA on Hypoglossal Nerve in PSD Mice

To observe spike counts of hypoglossal nerve, we found that each group (*n* = 6) was significantly different than normal (*p* < 0.01). Compared the model, spike counts of sham EA, EA (acute), and EA (chronic) were increased (*p* < 0.05, *p* < 0.01, and *p* < 0.01, separately). Compared with sham EA, EA (acute) and EA (chronic) were obvious increased (*p* < 0.01). Compared with EA (acute), EA (chronic) was decreased (*p* < 0.05) (Figure 3(f)). These results show that EA could excite the swallowing-related (hypoglossal) nerves, and chronic EA may inhibit peripheral nerves overexcitation and restore them to a nearly normal level.

### 3.5. The Effects of EA on MCV in PSD Mice

The hypoglossal nerve is one of the peripheral nerves innervating the swallowing of the mylohyoid muscle. We verified the effect of motor cortex injury on peripheral nerve discharges by stimulating the hypoglossal nerve trunk to induce the EMG of the muscle. It was found that the latency of action potential induced by dysphagia after stroke was significantly prolonged (*p* < 0.01) (Figures 4(c) and 4(g)), indicating that the motor conduction ability of hypoglossal nerve decreased significantly due to the M1 injury (*p* < 0.01) (Figure 4(h)). However, both acute and chronic EA treatment could obviously shorten the latency of the motor conduction (*p* < 0.01) (Figures 4(e)–4(g)), gradually increase the MNCV (*p* < 0.01) (Figure 4(h)), and recover the hypoglossal nerve function. It is inferred that dysphagia after stroke can affect the swallowing-related peripheral motor nerve, and EA could improve the conduction disorder caused by M1 injury, thus promoting the swallowing function recovery.

### 3.6. The Effects of EA on Swallowing Counts and EMG in PSD Mice

**Figure 5:**
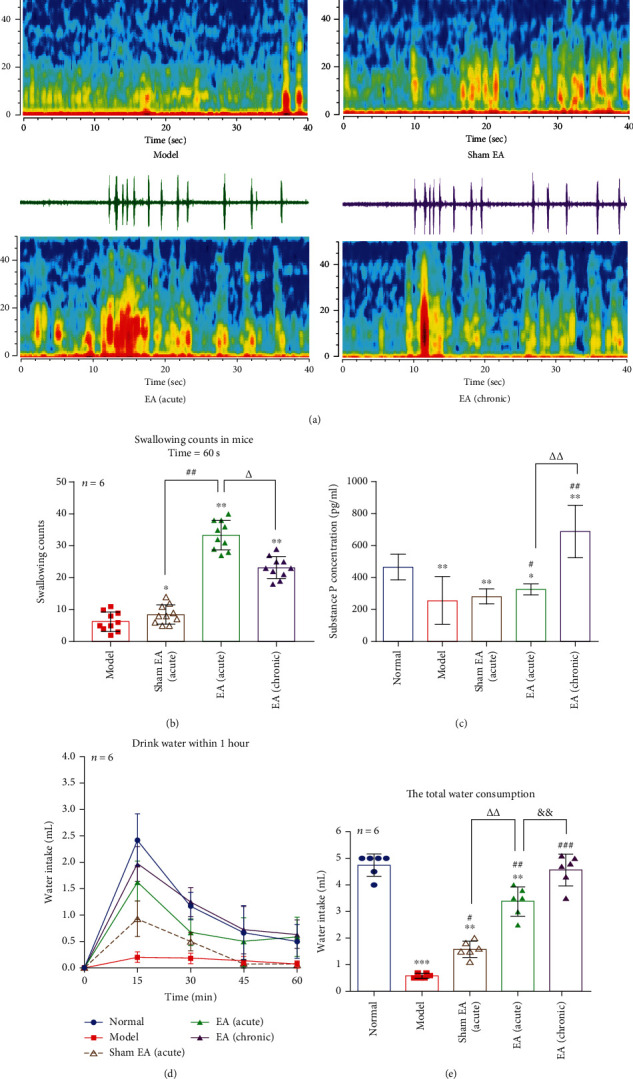
Electroacupuncture stimulation improves swallowing counts, SP release, and water intake in mice. (a) The changes of EMG and real-time spectrum in each group: EA (acute) > EA (chronic) > sham EA > model, time = 40 s, peak value = 0.02 mV. (b) Swallowing counts in each group: time = 60 s, compared with the model, ∗*p* < 0.05 in sham EA (acute) and ∗∗*p* < 0.01 in EA (acute) and EA (chronic). Compared with sham EA (acute), ^##^*p* < 0.01 in EA (acute). Compared with EA (acute), ^△^*p* < 0.05 in EA (chronic). (c) Substance P: compared with normal, ∗*p* < 0.05 in EA (acute) and ∗∗*p* < 0.01 in the model, sham EA (acute), and EA (chronic). Compared with the model, ^#^*p* < 0.05 in EA (acute), ^##^*p* < 0.01 in EA (chronic). EA (acute) vs. EA (chronic): ^△△^*p* < 0.01. (d) Mice drink water within 1 hour: normal > EA (chronic) > EA (acute) > sham EA (acute) > model. (e) Total water consumption: compared with normal, ∗∗∗*p* < 0.001 in the model and ∗∗*p* < 0.01 in sham EA (acute) and EA (acute). Compared with model, ∗*p* < 0.05 in sham EA (acute), ∗∗*p* < 0.01 in EA (acute), and ∗∗∗*p* < 0.001 in EA (chronic). Compared with sham EA (acute), ∗∗*p* < 0.01 in EA (acute). Compared with EA (acute), ∗∗*p* < 0.01 in EA (chronic).

Compared the EMG and real-time spectrum in each group (0.02 mV, time = 40 s), we found EMG, and power spectrum in the model and sham EA were lower than EA (acute) and EA (chronic). There was no little difference between EA (acute) and EA (chronic) in EMG, but power spectrum of EA (acute) was stronger than EA (chronic) (Figure 5(a)). In swallowing counts (*n* = 6, time = 60 s), the model was lower than any other groups (*p* < 0.05 and *p* < 0.01, separately). Compared with sham EA, EA (acute) was significantly increased (*p* < 0.01); compared with EA (acute), EA (chronic) was decreased (*p* < 0.05) (Figure 5(b)). These results illustrated that EA could enhance the swallowing-related muscle and increase the swallowing counts in PSD mice.

### 3.7. The Effects of EA on Drink Water and SP Release in PSD Mice

All of mice were deprived of water for 1 day. On day 2, each group was given drinking water *ad libitum* (*n* = 6), and we found the obvious changes in water intake at 15, 30, 45, and 60 min (Figure 5(d) and Table 2). In total water consumption, the water intake of model was the lowest than any other group (*p* < 0.001, *p* < 0.05, and *p* < 0.01, separately). Compare with sham EA, EA (acute) was significantly increased (*p* < 0.01); compared with EA (acute), EA (chronic) was obviously increased (Figure 5(e)). The SP concentration of the model was decreased compared with normal (*p* < 0.01). After EA (acute) and EA (chronic), the SP release was increased than the model (*p* < 0.05 and *p* < 0.01, separately) (Figure 5(c)). The results were confirmed that EA could promote the SP release and water intake and improve the swallowing function of PSD mice.

### 3.8. Swallowing Counts and Water Intake Are Correlated with Pyramidal Cell of M1 and Hypoglossal Nerve in PSD Mice

**Figure 6:**
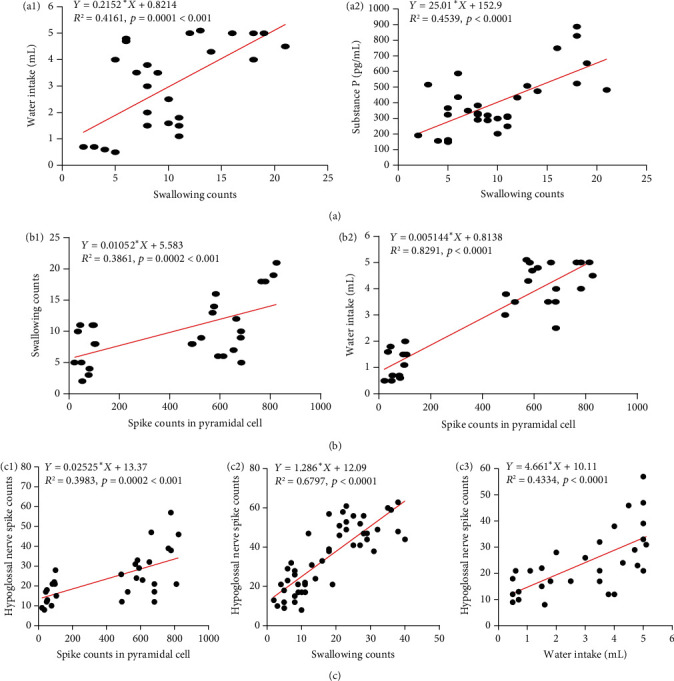
Linear correlation analysis of related-swallowing indicators in PSD mice. (a1) Swallowing counts were positively correlated with water intake, ∗∗∗*p* < 0.001, *r* = 0.6451. (a2) Swallowing counts were positively correlated with substance P, ∗∗∗*p* < 0.001, *r* = 0.6737. (b1) Spike counts in pyramidal cell were positively correlated with swallowing counts, ∗∗∗*p* < 0.001, *r* = 0.6213. (b2) Spike counts in pyramidal cell were positively correlated with water intake, ∗∗∗*p* < 0.001, *r* = 0.9105. (c1) Spike counts in pyramidal cell were positively correlated with hypoglossal nerve spike counts, ∗∗∗*p* < 0.001, *r* = 0.6311. (c2) Swallowing counts were positively correlated with hypoglossal nerve spike counts, ∗∗∗∗*p* < 0.0001, *r* = 0.8244. (c3) Water intake was positively correlated with hypoglossal nerve spike counts, ∗∗∗∗*p* < 0.0001, *r* = 0.6583.

We undertook linear correlation analysis of swallowing counts, spike counts in pyramidal cell, hypoglossal nerve spike counts, water intake, and SP in PSD mice by EA. First, we found the swallowing counts were positively correlated with water intake (*p* < 0.001, *r* = 0.6451) and SP (*p* < 0.001, *r* = 0.6737) (Figures 6(a1) and 6(a2)); it shows that the more swallowing counts, the more water intake and SP release in PSD mice. In addition, we found the pyramidal cell spike counts in M1 were positively correlated with swallowing counts (*p* < 0.001, *r* = 0.6213), water intake (*p* < 0.001, *r* = 0.9105), and hypoglossal spike counts (*p* < 0.001, *r* = 0.6311) (Figures 6(b1), 6(b2), and 6(c1)); it demonstrates that pyramidal cell in M1 affects hypoglossal nerve to increase the swallowing counts, which could promote SP release and recover the amount of water when the PSD mice swallowed. At last, hypoglossal nerve spike counts were positively correlated with swallowing counts (*p* < 0.0001, *r* = 0.8244) and water intake (*p* < 0.0001, *r* = 0.6583) (Figures 6(c2) and 6(c3)); it shows that swallowing-related peripheral nerves (hypoglossal) directly affect the swallowing counts and water intake in PSD mice. Based on the above correlation results, we conclude that EA could activate the swallowing-related pyramidal cell of the noninfarction area in M1, then directly affect hypoglossal nerve to neural regulate the swallowing counts and promote SP release, ultimately improved swallowing dysfunction in PSD mice.

## 4. Discussion

**Figure 7:**
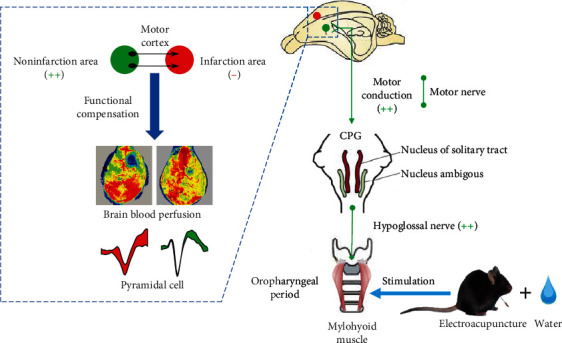
The EA stimulation involves in the motor neural control of motor cortex and hypoglossal nerves to improve the voluntary swallowing in poststroke dysphagia mice.

Stimulation the “Lianquan” acupoint (CV 23) by EA and feeding water induce the excitability of voluntary swallowing, in order to promote the motor cortex noninfarction neurons excitement and compensation the motor cortex function on the infarction side. The motor cortex on the noninfarction side transmitted motion excited to NTS and NA of the central pattern generator (CPG) that generate the swallowing reflex. At the same time, EA effects were exciting peripheral hypoglossal nerve, restoring the swallowing function of mylohyoid muscle, thereby to improve the dysphagia in oropharyngeal phase (Figure 7).

Dysphagia is characterized as being worse for liquids than solids [33]. M1 has a substantial role in the neural control the voluntary swallowing. Deglutition initiation and especially the oral phase of voluntary swallowing require the integrity of the motor areas of the cerebral cortex. We found that pyramidal cells were activated in noninfarction area of M1 by EA and that this action was associated with swallowing. In addition, stimulation of conception vessel (CV) 23 by EA could improve the blood flow in M1 and around deglutition-muscle groups, promote blood supplementation in tissue, and restore voluntary swallowing. Also, EA may reactivate peripheral nervous of voluntary swallowing, strengthen neural control of the hypoglossal nerve, release relevant neurotransmitters, recover voluntary swallowing gradually, and improve the PSD caused by unilateral M1 injury.

During voluntary swallowing, the cortex and subcortical areas play important roles in triggering and controlling the sequence of swallowing movements, especially in the oral phase [34]. Researchers have recognized the function of the cerebral cortex in swallowing control by observing patients with cortical dysphagia. However, the compensatory mechanism and functional recombination of dysphagia are not clear. Several experimental studies have suggested that the cerebral cortex has obvious hemisphere lateralization in response to swallowing. The locus of cortical control of swallowing lies anterior caudal to M1. The motor cortex is a crucial area for planning swallowing, and the oropharyngeal motor area is the central area which initiates swallowing [1, 3, 19, 35–40]. Therefore, patients with PSD may experience compensatory neurologic recombination in M1.

Consistent with this hypothesis, we found that the number and kind of neurons, spike counts in the noninfarction side of M1 in PSD mice reduced significantly (Figure 3(b); Table 1), so the compensatory mechanism could not be activated. After EA therapy immediately (acute) and EA for 3 days (chronic), pyramidal cells (which are the main projection neurons in the cerebral cortex) in the noninfarction side were activated to compensate the role of M1 in the infarction side (Figures 3(c) and 3(d)). Moreover, the activation of pyramidal cells promoted the increase in swallowing counts and water intake in PSD mice (Figures 6(b1) and 6(b2)). These data demonstrated that CV23 stimulation may activate the swallowing-related neurons (pyramidal cells) of M1 in the healthy side and improve voluntary swallowing.

The submental musculature offers the opportunity to assess M1 excitability during the different motor components of swallowing because it has a central role in the oral and pharyngeal phases of swallowing [30]. We recorded and compared the EMG of the mandibular swallowing muscles when drinking water in each group. Both acute and chronic EA treatment could stimulate the swallowing muscles in PSD mice, promote an increase in swallowing counts, and change water intake significantly (Figures 5(b), 5(d), and 5(e)). Simultaneously, stimulation of CV23 by EA could also improve the local blood flow of swallowing muscles, and blood perfusion in the noninfarction side in M1 was also increased significantly (Figure 2). The pharyngeal musculature is thought to be represented bilaterally but asymmetrically, suggesting hemispheric dominance in the motor control of these muscles [41]. For a healthy person, when swallowing water, the peak activation was occurred ≥12 s after the onset of swallowing, but, in patients with cortical dysphagia, peak activation occurs much later than 12 s [6]. We have observed this phenomenon in animal experiments. Hamdy et al. showed that to induce changes in excitation of the motor cortex with prolonged electrical stimulation of the pharynx for ≤15 min [3], the sensory input to the cortex must be manipulated, and compensatory recombination of the intact hemisphere must be undertaken to restore swallowing function in the pharynx [3, 28]. Hence, we undertook specific stimulation conduction of CV23 to strengthen PSD treatment using EA for 15 min.

Some studies have shown that acupuncture has a certain effect in this respect. Acupuncture at the corresponding acupoints can not only enhance nerve reflex but also promote muscle coordination and flexibility and achieve the purpose of improving swallowing [42–44]. “Lianquan” acupoint is between hyoid bone and thyroid cartilage, and the branches of swallowing nerve and hypoglossal nerve are located in its deep part. Deep needling can directly stimulate the swallowing muscle group and glossopharyngeal terminal nerve, and the reflex enhances the excitability of medulla oblongata, which is beneficial to the recovery of swallowing reflex arc. “Lianquan” acupoint is located in the control area of motor fibers of hypoglossal and swallowing nerves. Therefore, we choose “Lianquan” acupoint to observe the effect of poststroke dysphagia with theoretical basis.

The peripheral nervous receives signal from the central motor nerves and plays an important role in the voluntary swallowing. Pharyngeal phase starts by action of the pharyngeal plexus (which comprises the glossopharyngeal (IX), vagus (X), and accessory (XI) nerves) and the hypoglossal nerves on each side from the ansa cervicalis [45]. During swallowing, the hypoglossal (XII) nerves are responsible for the extrinsic and intrinsic muscles of the tongue. In addition, fibers from the cervical plexus in association with the hypoglossal nerve forms the ansa cervicalis will innervate the geniohyoid muscle [46, 47]. Jean suggested that the main motor nuclei involved in swallowing motor activity are the hypoglossal nucleus, and the nucleus ambiguus, hypoglossal, glossopharyngeal, and trigeminal nerves are the main motor nerves [48]. A lesion of the hypoglossal nerve can cause dysarthria, dysphagia, and tongue paralysis [49]. Sundman et al. suggested that the three mechanisms underlying dysphagia were delayed initiation of the swallowing reflex, impaired pharyngeal muscle function, and impaired coordination [50]; these symptoms of dysphagia are attributed to the damage of several central nuclei associated with swallowing [51, 52]. Related studies show that the loss of innervation of these muscles which the hyoid and anterior digastric muscles help to open the jaw and raise the hyoid during swallowing may be one of the causes of dysphagia in rats [53]. Therefore, there must be a certain relationship between the M1 responsible for initiating the swallowing reflex and the hypoglossal nerve innervating the digastric and lingual protrudor muscles in the neural regulation of dysphagia after stroke.

In this experiment, we found that the hypoglossal nerve activity has significantly decreased (*p* < 0.01, Figure 3(f)); the EMG of mylohyoid muscle was also inhibited (*p* < 0.01, Figures 5(a) and 5(b)), and the latency of evoked EMG activity by stimulation the hypoglossal nerve trunk was obviously prolonged (*p* < 0.01, Figures 4(c) and 4(g)) after the M1 injury, suggesting that the damage of motor cortex could affect the muscles innervated by hypoglossal nerve. Electrical stimulation of the hypoglossal nerve trunk cannot quickly induce action potentials, indicating that the central nervous regulation of swallowing is much greater than the peripheral local innervation. Acute or chronic EA could activate this nerve in PSD mice (*p* < 0.01, Figure 3(f)). The excitability of hypoglossal nerves was related to the spike counts of pyramidal cells in M1 on the healthy side (Figure 6(c1)), illustrating that EA promotes the excitation of M1, especially pyramidal cells, and then increases the discharge of hypoglossal nerve (*p* < 0.01), which has significantly shorten the latency of mylohyoid muscle and increases the MCV (*p* < 0.01); thus, the swallowing counts and water intake were further improved in dysphagia mice (Figures 3(f), 4(e)–4(h), 5(b), and 5(e)). It is revealed that EA could enhance the MCV of swallowing-related and help the body recover the swallowing function.

SP is a neurotransmitter that promotes the swallowing reflex in animals, and reduction of its secretion is related to inhibition of the swallowing reflex [54]. We showed that the SP concentration in serum was reduced dramatically in PSD mice, but after EA, it increased significantly (Figures 5(c) and 6(a2)). These data suggested that EA could promote SP release to improve swallowing function. Scholars have reported that SP can enhance swallow and cough reflexes and may also have a role in the response of the pharyngeal mucosa to local stimulation [55, 56]. Paul and colleagues showed that 78.6% of patients treated successfully by pharyngeal electrical stimulation showed a poststimulation increase in SP levels, whereas 88.9% of cases without clinical improvement of dysphagia had stable or decreased SP levels [57]. We postulate that pharyngeal electrical stimulation and EA stimulation have a close relationship in terms of the increase in SP levels. Thus, EA may trigger SP release, which results in peripheral sensitization of sensory neurons. This action would facilitate the motor swallowing response in the upstream swallowing network and enhance the excitability of the noninfarction side in M1 to aid recovery from PSD.

This study only observed the role of motor conduction and neural control of motor cortex-hypoglossal nerve by EA in PSD mice, but the ascending sensory conduction of EA stimulation is not involved. How EA involving sensory nerve conduction regulates the swallowing disorders may become the potential study in the future.

## Figures and Tables

**Table 1:** The different number of response neurons in each group of mice.

Groups (*n* = 6)	Model	Sham EA (acute)	EA (acute)	EA (chronic)	Normal
Interneuron	8	9	10	13	14
Pyramidal	2	4	8	10	15
Total	10	13	18	23	29

**Table 2:** Water intake changes at different time periods in each group of mice.

Groups (*n* = 6)	Model	Sham EA (acute)	EA (acute)	EA (chronic)	Normal
0 min	0	0	0	0	0
15 min	0.2 ± 0.089^∗∗^	0.993 ± 0.333^∗∗^/^##^	1.633 ± 0.383^∗∗^/^##^/^ΔΔ^	1.967 ± 0.327^∗^/^##^/^ΔΔ^	2.417 ± 0.491
30 min	0.183 ± 0.098^∗∗^	0.5 ± 0.179^∗∗^/^#^	0.667 ± 0.258^∗∗^/^##^	1.25 ± 0.274^##^/^ΔΔ^/^▲▲^	1.167 ± 0.258
45 min	0.133 ± 0.082^∗^	0.083 ± 0.075^∗^	0.5 ± 0.447	0.717 ± 0.449^#^/^Δ^	0.667 ± 0.516
60 min	0.067 ± 0.052^∗∗^	0.067 ± 0.052^∗∗^	0.583 ± 0.376^##^/^ΔΔ^	0.633 ± 0.273^##^/^ΔΔ^	0.5 ± 0.316

Notes: ^∗^*p* < 0.05 and ^∗∗^*p* < 0.01 versus normal; ^#^*p* < 0.05 and ^#^*p* < 0.01 versus model; ^∆^*p* < 0.05 and ^∆∆^*p* < 0.01 versus sham EA; ^▲▲^*P* < 0.01 versus EA (acute).

## Data Availability

The data used to support the findings of this study are included within the article.

## Abbreviations

CPG: Central pattern generator
CV: Conception vessel
EA: Electroacupuncture
EEG: Electroencephalogram
EMG: Electromyography
LFP: Local field potential
LSCI: Laser speckle contrast imaging
M1: Motor cortex 1
MCV: Motor conduction velocity
NA: Nucleus ambiguous
NTS: Nucleus of the solitary tract
PCA: Principal component analysis
PSD: Post-stroke dysphagia
SP: Substance P
SPKC: Spike continuous
VLM: Ventrolateral medulla.

## Supplementary Materials

Supplementary Materials Supplementary 1: PCA in each group, time = 300 s. (A) Normal: 5 units. (B) Model: 3 units. (C) Sham EA: 3 units. (D) EA acute: 3 units. (E) EA chronic: 3 units. Supplementary 2: autocorrelograms in each group, bin = 1 ms. (A) Normal: 2 interneurons (unit a and unit b) and 3 pyramidal cells (unit c, unit d, and unit e). (B) Model: 1 interneuron (unit a) and 2 pyramidal cells (unit b and unit c). (C) Sham EA: 1 interneuron (unit a) and 2 pyramidal cells (unit b and unit c). (D) EA acute: 1 interneuron (unit a) and 2 pyramidal cells (unit b and unit c). (E) EA chronic: 1 interneuron (unit a) and 2 pyramidal cells (unit b and unit c). Supplementary 3: the motor cortex neuron changes of noninfarction area were recorded by multichannel electrophysiology *in vivo*. (A) Multichannel recording electrode: 2 × 4 + 1 matrix electrode implantation. (B) Electrode recording site: bregma: −0.16 mm; left: 1 mm; depth: 1 mm. (C) Photograph showing the site of the recording electrode: M1, bar = 500 *μ*m. (D) Normal: recording 5 units. Normal-before vs. normal-after: time = 5 min in each phase, interval = stop recording 15 min. Interneuron vs. pyramidal: before = 29.65% vs. 70.35%, after = 35.1% vs. 64.9%. (E) Model: recording 3 units. Before sham EA vs. after sham EA: time = 5 min in each phase, interval = lack of electric stimulation 15 min. Interneuron vs. pyramidal: before sham EA = 17.01% vs. 82.99%, after sham EA = 18.25% vs. 81.75%. (F) Model: recording 3 units. Before EA vs. after EA (acute) vs. after EA (chronic): time = 5 min in each phase, interval 1 = EA stimulation 15 min, interval 2 = EA stimulation for 3 days interneuron vs. pyramidal: before EA = 15.73% vs. 84.27%, after EA (acute) = 33.62% vs. 66.38%, and after EA (chronic) = 34.6% vs. 65.4%. Supplementary 4: the hypoglossal nerve was recorded *in vivo*.

## References

[1] Hamdy S., Aziz Q., Thompson D. G., Rothwell J. C. (2001). Physiology and pathophysiology of the swallowing area of human motor cortex. *Neural Plasticity*.

[2] Hamdy S., Mikulis D. J., Crawley A. (1999). Cortical activation during human volitional swallowing: an event-related fMRI study. *The American Journal of Physiology*.

[3] Hamdy S., Rothwell J. C., Aziz Q., Thompson D. G. (2000). Organization and reorganization of human swallowing motor cortex: implications for recovery after stroke. *Clinical Science*.

[4] Hamdy S., Rothwell J. C., Brooks D. J., Bailey D., Aziz Q., Thompson D. G. (1999). Identification of the cerebral loci processing human swallowing with H_2_^15^O PET activation. *Journal of Neurophysiology*.

[5] Mourao A. M., Lemos S. M., Almeida E. O., Vicente L. C., Teixeira A. L. (2016). Frequency and factors associated with dysphagia in stroke. *Codas*.

[6] Yang H., Ang K. K., Wang C., Phua K. S., Guan C. (2016). Neural and cortical analysis of swallowing and detection of motor imagery of swallow for dysphagia rehabilitation – a review. *Progress in Brain Research*.

[7] Cohen D. L., Roffe C., Beavan J. (2016). Post-stroke dysphagia: a review and design considerations for future trials. *International Journal of Stroke*.

[8] Falsetti P., Acciai C., Palilla R. (2009). Oropharyngeal dysphagia after stroke: incidence, diagnosis, and clinical predictors in patients admitted to a neurorehabilitation unit. *Journal of Stroke and Cerebrovascular Diseases*.

[9] Umay E. K., Unlu E., Saylam G. K., Cakci A., Korkmaz H. (2013). Evaluation of dysphagia in early stroke patients by bedside, endoscopic, and electrophysiological methods. *Dysphagia*.

[10] Chen S. Y., Chie W. C., Lin Y. N., Chang Y. C., Wang T. G., Lien I. N. (2004). Can the aspiration detected by videofluoroscopic swallowing studies predict long-term survival in stroke patients with dysphagia?. *Disability and Rehabilitation*.

[11] Ding R., Logemann J. A. (2000). Pneumonia in stroke patients: a retrospective study. *Dysphagia*.

[12] Teasell R., Foley N., Fisher J., Finestone H. (2002). The incidence, management, and complications of dysphagia in patients with medullary strokes admitted to a rehabilitation unit. *Dysphagia*.

[13] Matsuo K., Palmer J. B. (2008). Anatomy and physiology of feeding and swallowing: normal and abnormal. *Physical Medicine and Rehabilitation Clinics of North America*.

[14] Mckeown M. J., Torpey D. C., Gehm W. C. (2002). Non-invasive monitoring of functionally distinct muscle activations during swallowing. *Clinical Neurophysiology*.

[15] You H., Hu S., Ye Q. (2018). Role of 5-HT1A in the nucleus of the solitary tract in the regulation of swallowing activities evoked by electroacupuncture in anesthetized rats. *Neuroscience Letters*.

[16] Furlong P. L., Hobson A. R., Aziz Q. (2004). Dissociating the spatio-temporal characteristics of cortical neuronal activity associated with human volitional swallowing in the healthy adult brain. *NeuroImage*.

[17] Michou E., Hamdy S. (2009). Cortical input in control of swallowing. *Otolaryngology and Head and Neck Surgery*.

[18] Li S., Luo C., Yu B. (2009). Functional magnetic resonance imaging study on dysphagia after unilateral hemispheric stroke a preliminary study. *Journal of Neurology, Neurosurgery, and Psychiatry*.

[19] Yuan X. D., Zhou L. F., Wang S. J. (2015). Compensatory recombination phenomena of neurological functions in central dysphagia patients. *Neural Regeneration Research*.

[20] Bahceci K., Umay E., Gundogdu I., Gurcay E., Ozturk E., Alıcura S. (2017). The effect of swallowing rehabilitation on quality of life of the dysphagic patients with cortical ischemic stroke. *Iranian journal of neurology*.

[21] Li L., Deng K., Qu Y. (2018). Acupuncture treatment for post-stroke dysphagia: an update meta-analysis of randomized controlled trials. *Chinese Journal of Integrative Medicine*.

[22] Cai H., Ma B., Gao X., Gao H. (2015). Tongue acupuncture in treatment of post-stroke dysphagia. *International Journal of Clinical and Experimental Medicine*.

[23] Wang Y., Shen J., Wang X. M. (2012). Scalp acupuncture for acute ischemic stroke: a meta-analysis of randomized controlled trials. *Evidence-based Complementary and Alternative Medicine*.

[24] Goyal R. K., Padmanabhan R., Sang Q. (2001). Neural circuits in swallowing and abdominal vagal afferent-mediated lower esophageal sphincter relaxation. *The American Journal of Medicine*.

[25] Shi J., Ye Q., Zhao J. (2019). EA promotes swallowing via activating swallowing-related motor neurons in the nucleus ambiguus. *Brain Research*.

[26] Ye Q., Liu C., Shi J. (2019). Effect of electro-acupuncture on regulating the swallowing by activating the interneuron in ventrolateral medulla (VLM). *Brain Research Bulletin*.

[27] Doeltgen S. H., Dalrymple-Alford J., Ridding M. C., Huckabee M. L. (2010). Differential effects of neuromuscular electrical stimulation parameters on submental Motor-Evoked Potentials. *Neural Repair*.

[28] Hamdy S., Rothwell J. C., Aziz Q., Singh K. D., Thompson D. G. (1998). Long-term reorganization of human motor cortex driven by short-term sensory stimulation. *Nature Neuroscience*.

[29] Humbert I. A., Joel S. (2012). Tactile, gustatory, and visual biofeedback stimuli modulate neural substrates of deglutition. *NeuroImage*.

[30] Sebastian H. D., Michael C. R., John D. A., Huckabee M. L. (2011). Task-dependent differences in corticobulbar excitability of the submental motor projections: implications for neural control of swallowing. *Brain Research Bulletin*.

[31] Michael S., Sebastian J., Guido S. (2002). Non-invasive induction of focal cerebral ischemia in mice by photothrombosis of cortical microvessels: characterization of inflammatory responses. *Journal of neuroscience methods*.

[32] Pang G., Zhang G. L. (2014). Subcellular localization of serotonin 2A receptor in dorsal hippocampal CA1 area and its effect on neuronal firing. *Chinese Pharmacological Bulletin*.

[33] Lancaster J. (2015). Dysphagia: its nature, assessment and management. *British Journal of Community Nursing*.

[34] Ertekin C. (2011). Voluntary versus spontaneous swallowing in man. *Dysphagia*.

[35] Forman S. D., Cohen J. D., Fitzgerald M., Eddy W. F., Mintun M. A., Noll D. C. (1995). Improved assessment of significant activation in functional magnetic resonance imaging (fMRI): use of a cluster-size threshold. *Magnetic Resonance in Medicine*.

[36] Lacourse M. G., Orr E. L., Cramer S. C., Cohen M. J. (2005). Brain activation during execution and motor imagery of novel and skilled sequential hand movements. *NeuroImage*.

[37] Komisaruk B. R., Mosier K. M., Liu W. C. (2002). Functional localization of brainstem and cervical spinal cord nuclei in humans with fMRI. *American Journal of Neuroradiology*.

[38] Lotze M., Montoya P., Erb M. (1999). Activation of cortical and cerebellar motor areas during executed and imagined hand movements: an fMRI study. *Journal of Cognitive Neuroscience*.

[39] Saad Z. S., Chen G., Reynolds R. C. (2006). Functional imaging analysis contest (FIAC) analysis according to AFNI and SUMA. *Human brain mapping*.

[40] Szameitat A. J., Shen S., Sterr A. (2007). Motor imagery of complex everyday movements: an fMRI study. *NeuroImage*.

[41] Mistry S., Verin E. S., Singh S. (2007). Unilateral suppression of pharyngeal motor cortex to repetitive transcranial magnetic stimulation reveals functional asymmetry in the hemispheric projections to human swallowing. *The Journal of Physiology*.

[42] Chen Y. X. (2018). Therapeutic effect of acupuncture combined with swallowing training on patients with dysphagia after cerebral infarction. *Chinese Journal of Modern Drug Application*.

[43] Wang L., Pei Y. (2016). Acupuncture for dysphagia after stroke. *Journal of Practical Traditional Chinese Internal Medicine*.

[44] Yang F. X., Chen L. (2017). A clinical study of tongue needle combined with catheter balloon dilatation in the treatment of dysphagia after stroke. *Shanghai journal of acupuncture and moxibustion*.

[45] Costa M. M. B. (2018). Neural control of swallowing. *Arquivos de Gastroenterologia*.

[46] Chusid J. G. (1985). *Neuroanatomiacorrelativa e Neurologiafuncional*.

[47] Guyton A. C., Hall J. E. (2011). *Textbook of Medical Physiology*.

[48] Jean A. (2001). Brain stem control of swallowing: neuronal network and cellular mechanisms. *Physiological Reviews*.

[49] Meng S., Reissig L. F., Tzou C. H., Meng K., Grisold W., Weninger W. (2016). Ultrasound of the hypoglossal nerve in the neck: visualization and initial clinical experience with patients. *American Journal of Neuroradiology*.

[50] Sundman E., Witt H., Olsson R., Ekberg O., Kuylenstierna R., Eriksson L. I. (2000). The incidence and mechanisms of pharyngeal and upper esophageal dysfunction in partially paralyzed humans: pharyngeal videoradiography and simultaneous manometry after atracurium. *Anesthesiology*.

[51] Lever T. E., Gorsek A., Cox K. T. (2009). An animal model of oral dysphagia in amyotrophic lateral sclerosis. *Dysphagia*.

[52] Lever T. E., Simon E., Cox K. T. (2010). A mouse model of pharyngeal dysphagia in amyotrophic lateral sclerosis. *Dysphagia*.

[53] Travers J. B., Jackson L. M. (1992). Hypoglossal neural activity during licking and swallowing in the awake rat. *Journal of Neurophysiology*.

[54] Nakashima T., Hattori N., Okimoto M., Yanagida J., Kohno N. (2011). Nicergoline improves dysphagia by upregulating substance P in the elderly. *Medicine*.

[55] Imoto Y., Kojima A., Osawa Y., Sunaga H., Fujieda S. (2011). Cough reflex induced by capsaicin inhalation in patients with dysphagia. *Acta Oto-Laryngologica*.

[56] Jin Y., Sekizawa K., Fukushima T., Morikawa M., Nakazawa H., Sasaki H. (1994). Capsaicin desensitization inhibits swallowing reflex in guinea pigs. *American Journal of Respiratory and Critical Care Medicine*.

[57] Paul M., Sonja S. K., Stefan B. (2017). Increase of substance P concentration in saliva after pharyngeal electrical stimulation in severely dysphagic stroke patients – an indicator of decannulation success?. *Neurosignals*.

