# Supplementary material for: Electroacupuncture Involved in Motor Cortex and Hypoglossal Neural Control to Improve Voluntary Swallowing of Poststroke Dysphagia Mice
Source: Neural Plast. 2020 Sep 27;2020:8857543. doi: 10.1155/2020/8857543 (PMC7537716; doi:10.1155/2020/8857543)
Supplement: Supplementary Materials — Supplementary 1: PCA in each group, time = 300 s. (A) Normal: 5 units. (B) Model: 3 units. (C) Sham EA: 3 units. (D) EA acute: 3 units. (E) EA chronic: 3 units. Supplementary 2: autocorrelograms in each group, bin = 1 ms. (A) Normal: 2 interneurons (unit a and unit b) and 3 pyramidal cells (unit c, unit d, and unit e). (B) Model: 1 interneuron (unit a) and 2 pyramidal cells (unit b and unit c). (C) Sham EA: 1 interneuron (unit a) and 2 pyramidal cells (unit b and unit c). (D) EA acute: 1 interneuron (unit a) and 2 pyramidal cells (unit b and unit c). (E) EA chronic: 1 interneuron (unit a) and 2 pyramidal cells (unit b and unit c). Supplementary 3: the motor cortex neuron changes of noninfarction area were recorded by multichannel electrophysiology in vivo. (A) Multichannel recording electrode: 2 × 4 + 1 matrix electrode implantation. (B) Electrode recording site: bregma: −0.16 mm; left: 1 mm; depth: 1 mm. (C) Photograph showing the site of the recording electrode: M1, bar = 500 μm. (D) Normal: recording 5 units. Normal-before vs. normal-after: time = 5 min in each phase, interval = stop recording 15 min. Interneuron vs. pyramidal: before = 29.65% vs. 70.35%, after = 35.1% vs. 64.9%. (E) Model: recording 3 units. Before sham EA vs. after sham EA: time = 5 min in each phase, interval = lack of electric stimulation 15 min. Interneuron vs. pyramidal: before sham EA = 17.01% vs. 82.99%, after sham EA = 18.25% vs. 81.75%. (F) Model: recording 3 units. Before EA vs. after EA (acute) vs. after EA (chronic): time = 5 min in each phase, interval 1 = EA stimulation 15 min, interval 2 = EA stimulation for 3 days interneuron vs. pyramidal: before EA = 15.73% vs. 84.27%, after EA (acute) = 33.62% vs. 66.38%, and after EA (chronic) = 34.6% vs. 65.4%. Supplementary 4: the hypoglossal nerve was recorded in vivo. [file 8857543.f1.docx]

**Supplementary Figures**

**
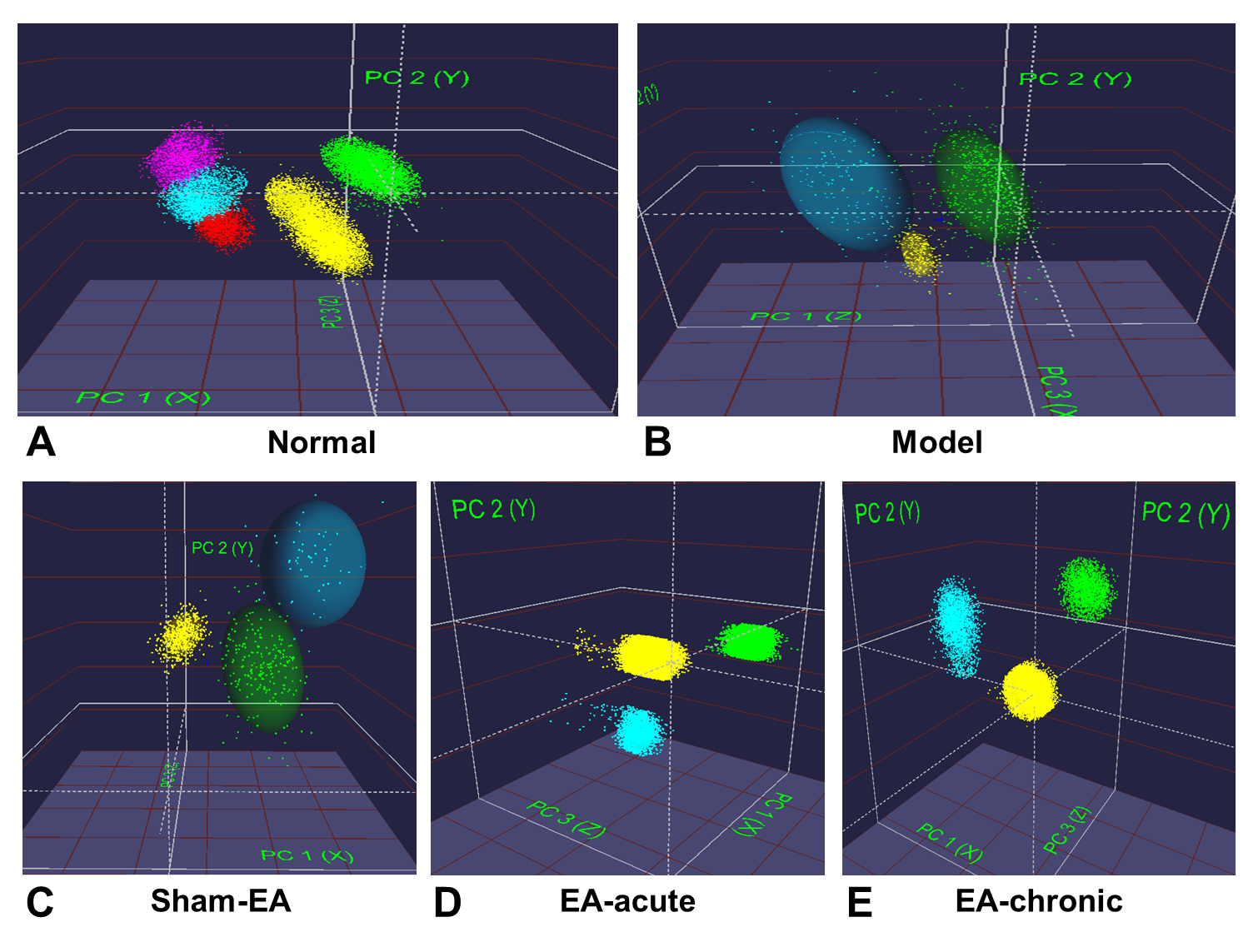
**

**Supplementary 1. PCA in each group, time = 300 s. (A)** Normal: 5 units; **(B)** Model: 3 units; **(C)** Sham EA: 3 units; **(D)** EA acute: 3 units; **(E)** EA chronic: 3 units.

**
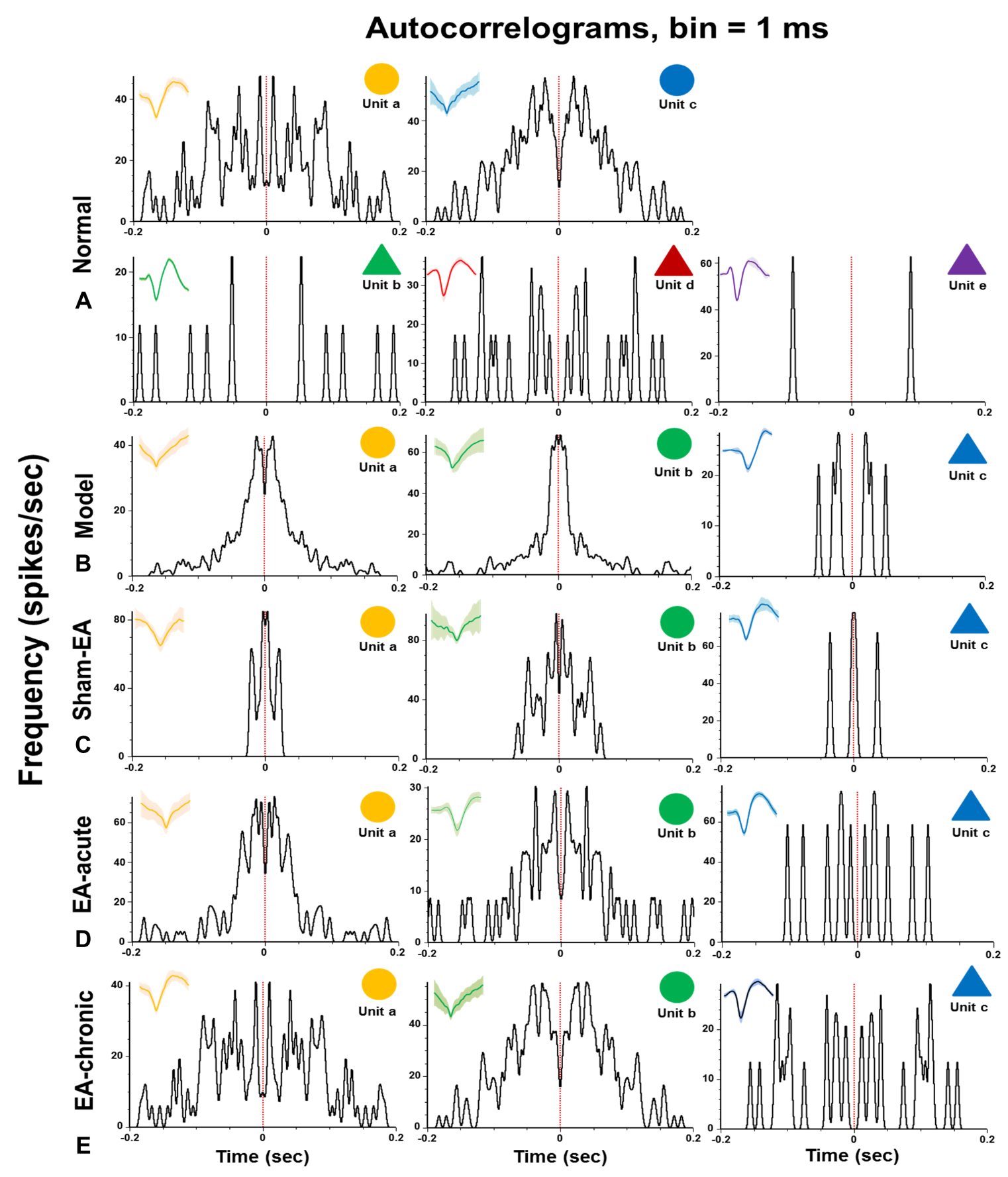
**

**Supplementary 2.Autocorrelograms in each group, bin = 1 ms. (A)** Normal: 2 interneurons (Unit a & Unit b) and 3 pyramidal cells (Unit c, Unit d & Unit e); **(B)** Model: 1 interneuron (Unit a) and 2 pyramidal cells (Unit b & Unit c); **(C)** sham-EA: 1 interneuron (Unit a) and 2 pyramidal cells (Unit b & Unit c); **(D)** EA acute: 1 interneuron (Unit a) and 2 pyramidal cells (Unit b & Unit c); **(E)** EA chronic: 1 interneuron (Unit a) and 2 pyramidal cells (Unit b & Unit c).

**
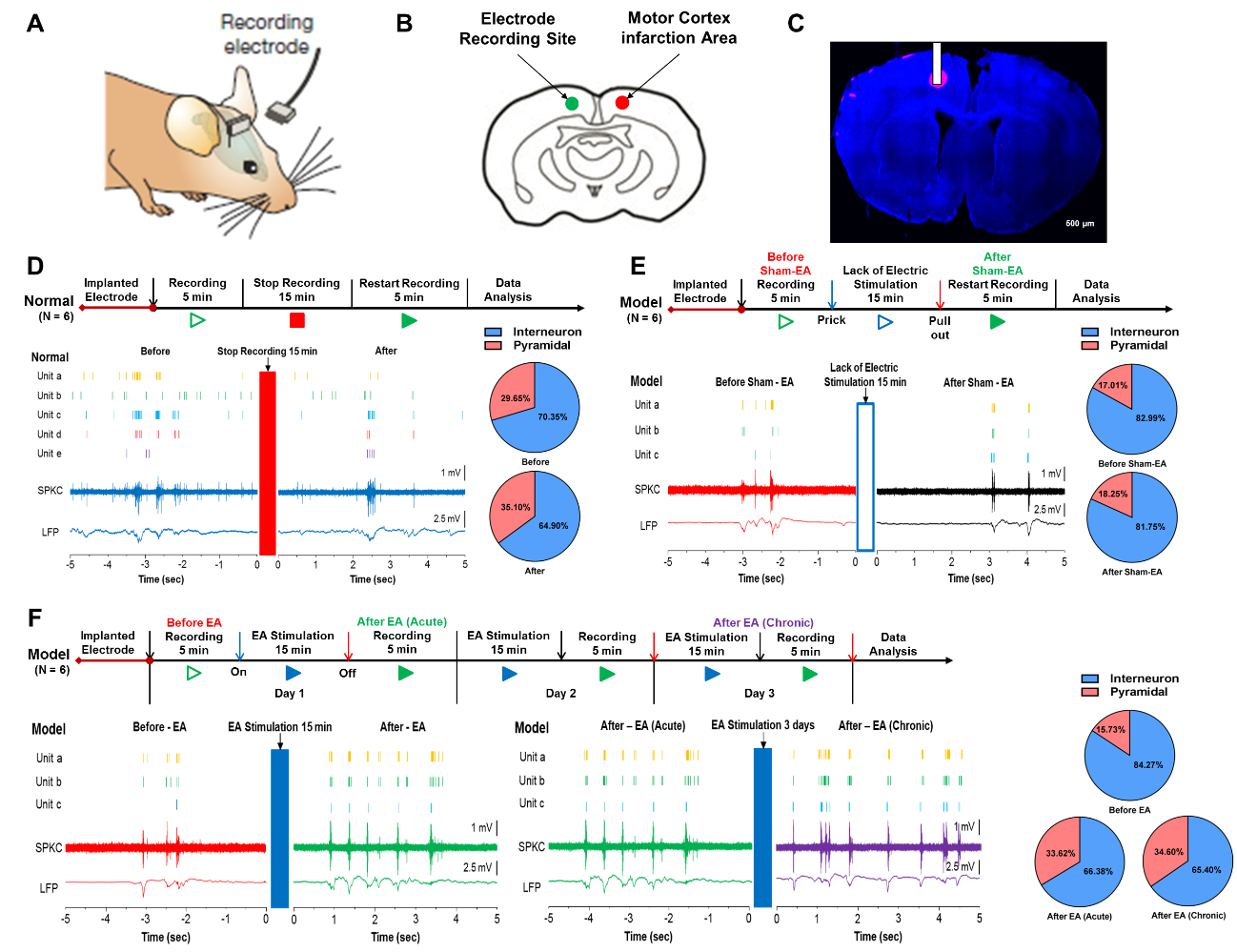
**

**Supplementary 3.The motor cortex neurons changes of non-infarction area were recorded by multi-channel electrophysiology *in vivo*. (A)** Multi-channel recording electrode: 2 X 4 + 1 matrix electrode implantation. **(B)** Electrode recording site: Bregma: −0.16 mm; Left: 1 mm; Depth: 1 mm. **(C)** Photograph showing the site of the recording electrode: M1, bar = 500 μm. **(D)** Normal: Recording 5 units. Normal-before vs. normal-after: time = 5 min in each phase, interval = stop recording 15 min. Interneuron vs. pyramidal: before = 29.65 % vs. 70.35 %, after = 35.1 % vs. 64.9 %. **(E)** Model: Recording 3 units. Before sham-EA vs. after sham-EA: time = 5 min in each phase, interval = lack of electric stimulation 15 min. Interneuron vs. pyramidal: before sham-EA = 17.01 % vs. 82.99 %, after sham-EA = 18.25 % vs. 81.75 %. **(F)** Model: Recording 3 units. Before EA vs. after EA (Acute) vs. after EA (Chronic): time = 5 min in each phase, interval 1= EA stimulation 15 min, interval 2 = EA stimulation for 3 days Interneuron vs. pyramidal: before EA = 15.73 % vs. 84.27 %, after EA (Acute) = 33.62 % vs. 66.38 %, after EA (Chronic) = 34.6 % vs. 65.4 %.

**
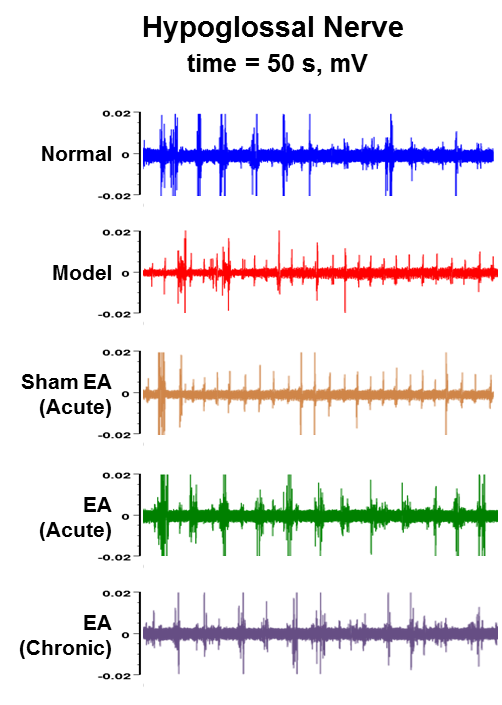
**

**Supplementary 4.The hypoglossal nerve was recorded *in vivo*.**
